# Tomographic Aspects of Advanced Active Pulmonary Tuberculosis and Evaluation of Sequelae following Treatment

**DOI:** 10.1155/2017/9876768

**Published:** 2017-02-05

**Authors:** Rafael Barcelos Capone, Domenico Capone, Thiago Mafort, Roberto Mogami, Rosana de Souza Rodrigues, Miriam Menna Barreto, Rogerio Rufino

**Affiliations:** ^1^Department of Medical Sciences, Rio de Janeiro State University, Rio de Janeiro, RJ, Brazil; ^2^Pulmonology and Radiology Services, Rio de Janeiro State University, Rio de Janeiro, RJ, Brazil; ^3^Federal University of Rio de Janeiro, Rio de Janeiro, RJ, Brazil

## Abstract

*Objectives.* To evaluate tomographic changes in pulmonary tuberculosis (TB), degree of agreement among three radiologists regarding tomographic diagnoses, and sequelae following treatment.* Methods.* Cross-sectional and descriptive study of 74 TB patients confirmed by sputum culture and chest computed tomography before (CT1) and 6 months after (CT2) drug therapy. Results were performed by three radiologists blinded to clinical and laboratory results.* Results.* Main findings in CT1 included nodules indicating the presence of a tree-in-bud pattern in 93% of cases, ill-defined nodules in 84% of cases, consolidation in 77% of cases, architectural distortion in 71% of cases, cavitary lesions in 62% of cases, and ground glass opacities in 37% of cases. Airway involvement, characterized by increased thickness and dilatation of the bronchial walls, occurred in 93% of cases. Pleural involvement occurred in 54%. There was an agreement on active TB among the three radiologists in 85% of cases. The results in CT2 indicated the presence of architectural distortion in 91% of cases and cylindrical bronchiectasis in 86%.* Conclusions.* The study established a tomographic pattern for diagnosis of active TB characterized by the presence of airway nodules, consolidation, architectural distortion, and cavitary lesions, and an almost complete degree of agreement (Kappa) was observed among the radiologists (0.85). CT after treatment assists in defining the cure.

## 1. Introduction

Tuberculosis (TB) is one of the few diseases whose etiology has been known for over a century. Although effective treatments are available, it remains a relevant and global health problem. Approximately one-third of the world population is infected with* Mycobacterium tuberculosis* (MTB), particularly in developing countries. Early diagnosis plays an important role in the control of TB, and, at present, it is the most effective strategy for interfering in the transmission chain [1].

Multiple factors are responsible for delayed diagnosis. The loss of sensitivity of the methods used to detect alcohol acid-resistant bacilli (AARB) in sputum is one of these factors. Sputum smear microscopy is the most widespread method for diagnosis of TB. However, its sensitivity varies in one-third to two-thirds of the cases, and a significant number of patients have negative AARB test results. Sputum culture remains the gold standard for diagnosis, but the results can take 4 to 6 weeks, which limits prompt decision-making. New tests have emerged to aid in TB diagnosis, including GeneXpert. This test is being implemented in many parts of the world, including Brazil, but it does not diagnose active disease [1–3]. Nonetheless, it indicates the possibility of TB occurrence and even resistance to rifampicin. For confirmation of TB, MTB culture is essential.

Imaging methods have long been used to aid in TB diagnosis. Despite its low specificity, chest radiography is still an extremely valuable technique used in the initial management of patients with respiratory symptoms. Moreover, chest radiography is a valuable complement to physical examination, as it can detect multiple clinical changes and it is essential in differential diagnosis [4].

Chest computed tomography (CT) is more sensitive than radiography in detecting initial clinical changes. In addition, CT can distinguish active lesions from residual lesions in most cases, accurately assess the extent of the disease, and determine a diagnostic standard based on the major changes observed. Therefore, although this method is not highly specific for TB diagnosis, CT adds diagnostic information and is a particularly valuable method for patients with suspected TB and negative AARB test results, allowing adequate therapeutic decision-making while waiting for sputum culture results to confirm TB [3–7].

The present study aimed to describe the main computed tomography findings, to assess the extent of active pulmonary TB before treatment (CT1) and 6 months after treatment (CT2), and to establish the degree of agreement among three radiologists with proven experience in CT for TB diagnosis.

## 2. Methods

This prospective and descriptive study evaluated patients with proven pulmonary TB who were subjected to chest CT before drug therapy (CT1) and after drug therapy (CT2) at the Tuberculosis Center of the Pulmonology Department of the State University of Rio de Janeiro between 2012 and 2014. The present study adhered to the following guidelines: medical and nursing interviews, including the collection of data on identification, biometrics, profession, education, symptoms, duration of symptoms (defined as the period between the onset of symptoms reported by the patient and the first consultation in the clinic), and the period between the completion of the first CT and initiation of treatment. The patients followed outpatient routines, including blood tests, collection of two AARB samples, Koch's bacillus (KB) culture, and imaging tests. Some patients had negative AARB test results and chest CT results obtained at another institution during the investigation. All patients were respiratory symptomatic. After treatment, chest CT2 was performed and compared with CT1. CT2 was performed within 7 days of the completion of drug therapy for TB. The TB treatment used was recommended by the Ministry of Health of Brazil and included rifampicin, isoniazid, pyrazinamide, and ethambutol for a period of 6 months. All patients signed a free and informed consent form, and the study was approved by the Research Ethics Committee of HUPE/UERJ under protocol number 70308/2012.

The inclusion criteria were clinical suspicion of TB with negative AARB test results, diagnostic confirmation by MTB culture in sputum, and performance of CT1 and CT2. Patients excluded from the study were those with HIV, with diabetes mellitus, using biopharmaceuticals for rheumatic disease, with previous TB (relapse), with non-TB mycobacteriosis, who were pregnant, with sputum culture and/or bronchoalveolar lavage negative for TB, and who did not undergo CT2. The CT examinations included standard chest scans using a 64-channel scanner (model Brilliance 40, Philips Medical Systems, Cleveland, OH, USA) complemented with images obtained with a high-resolution technique and high-frequency algorithms, 512 × 512 matrix, and window and center with variation between 1000 and 1300 Hounsfield units (HU) and between −600 and −700 HU, respectively. The CT1 scans were independently analyzed by three radiologists specializing in thoracic diseases and blind to the clinical and laboratory results. The main parenchymal, pleural, and mediastinal changes were described following the guidelines of the glossary of terms for CT from the Fleischner Society [8]. After the description of the changes and documentation in a specific form, each radiologist was asked whether the tomographic diagnosis was consistent with active pulmonary TB, and, in negative cases, they were requested to provide an alternative diagnosis. Among the CT scans of TB patients, 20 cases of non-TB lung disease, requiring a differential diagnosis with TB, were included at random. Kappa concordance testing was conducted among the radiologists in the analysis of CT1 and of the 20 non-TB cases. Findings were expressed in percentages or averages.

## 3. Results

From the 94 patients with clinical suspicion and imaging results consistent with pulmonary TB, 10 patients were excluded because of negative BK sputum culture results and another 10 for were excluded for not having undergone a CT2 scan after 6 months of treatment. Twenty chest CT scans presenting other diseases (sarcoidosis, leptospirosis, cancer, lymphoma, pneumonia, and cryptococcosis) were also analyzed at CT1 by three radiologists.

Forty-nine TB patients (66%) were Caucasian, and, at the time of the study, 61 patients (82.4%) had attended elementary school. Eighteen patients had a history of smoking (past and present).

Of the 74 patients with active pulmonary TB aged between 18 and 77 years (mean of 47.5 years), 34 were women (46%) and 40 were men (54%). The main clinical manifestations were cough (78%), weakness (74%), weight loss (69%), fever (66%), evening fever (50%), night sweats (48%), dyspnea and chest pain (40%), and hemoptoic expectoration (20%).

The approximate length of symptoms varied between 0 and 730 days, with an average of 105.3 days. The period between completion of CT1 and initiation of treatment varied between 0 and 892 days, with a mean period of 51.8 days. In CT1, there was agreement in diagnosis of active TB among three radiologists in 85% of cases and disagreement in 11 cases (15%). With regard to 20 CT scans of non-TB diseases included in the study, diagnostic agreement was observed among all three radiologists in 65% of cases and among two radiologists in 85% of cases.

**Figure 1:**
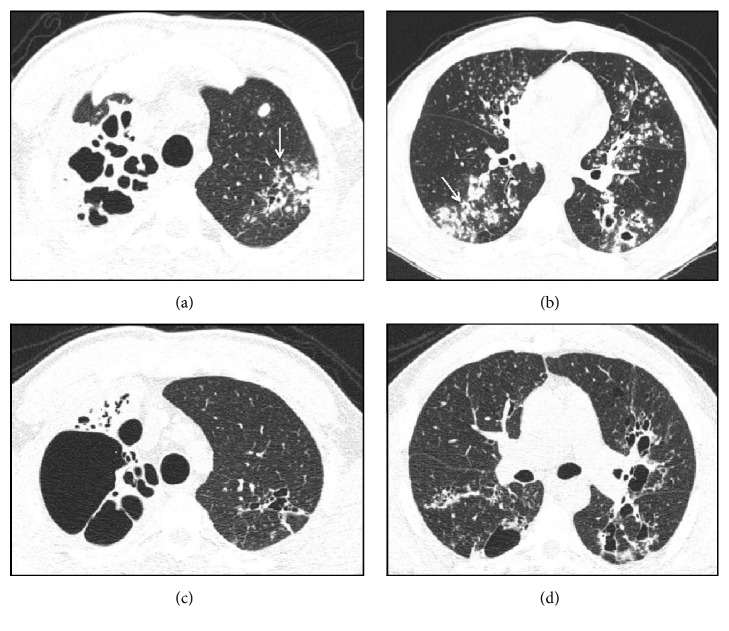
Computed tomography findings in TB. Axial views ((a) and (b)) before treatment indicating the presence of extensive areas of parenchymal damage characterized by cavitary lesions and bronchiectasis on the right in addition to larger grouped nodules and airway nodules configuring the tree-in-bud pattern, with some resembling a clover on the left (arrows). (c) and (d) represent sections obtained after treatment indicating the presence of gross residual changes characterized by bubbles and bronchiectasis intermingled with areas of fibrosis.

Regarding severity of the disease, multiple bilateral involvement was observed in 52 patients (70.3%) (Table 1 and Figure 1).

Tree-in-bud pattern was observed in 69 cases (93%) and was always associated with other types of injury, including consolidation in 57 cases (77%) and cavitary lesions in 46 cases (62%). Consolidations predominated in the upper lobes and occurred on the left lobe in 32 cases (56%) and on the right lobe in 22 cases (38%). Cavitary lesions predominated in upper lobes and occurred in upper left lobe in 22 cases (47.8%) and in upper right lobe in 15 cases (32.0%). The left and right lungs were affected separately in 12 cases (16.2%) and 10 cases (13.5%), respectively. Involvement of a single lobe was observed in 21% of cases, two lobes were involved in 21% of cases, three lobes in 19% of cases, and more than three lobes in 37% of cases. The upper lobe was affected in 72% of cases, the lingular and middle lobes were affected in 36% and 33% of cases, respectively, the right lower lobe was affected in 40% of cases, and the left lower lobe was affected in 60% of cases. Analysis by segment indicated that the most affected segments were the apical and posterior segments of the upper lobes in 63% and 66% of cases, respectively, and the apical segments of the lower lobes (41% of cases involving the right lobe and 52% of cases involving the left lobe).

**Figure 2:**
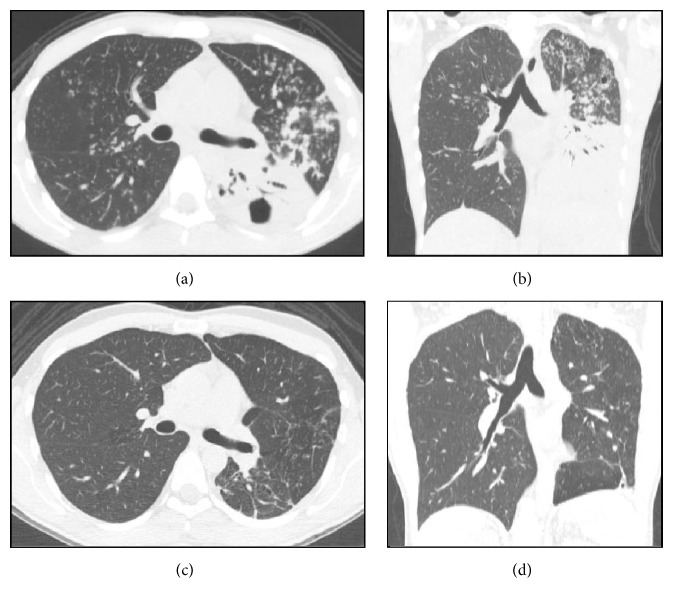
Computed tomography findings in TB. Axial view (a) and coronal reformatting (b) of TB before treatment, recorded in parenchymal window, indicating extensive consolidations intermingled with air bronchograms in the left lower lobe, associated with cavitary lesions and draining bronchus, confluent opacities, and bronchogenic dissemination characterized by airway nodules configuring the tree-in-bud pattern observed in the left upper lobe and right lung. Additionally, note the airway changes represented by increased wall thickness and bronchial dilation in the left upper lobe. (c) and (d) show the structural changes observed after treatment, characterized by volumetric reduction of the left lung, architectural distortions, thick bands of fibrous aspect on the left base, and bronchial changes in the left upper lobe.

The analysis of CT2 followed the same methodology. There was an absolute predominance of airway changes characterized by cylindrical bronchiectasis, pulmonary parenchyma distortions, and 1–3 cm diameter nodules (Table 2 and Figure 2).

Other features of CT1 included parenchymal calcifications in 32 cases (43%) and pleural abnormalities in 40 cases (54%), and these changes were characterized as pleural thickness in 22 cases (55%) and effusion in 18 cases (45%). Pleural effusion was bilateral in only two cases (5%). Small-volume pericardial effusion was observed in four (5.4%) patients. Regarding lymph node involvement, 13 (17.5%) patients exhibited calcified lymph nodes.

Lymph nodes with a diameter greater than 1 cm were observed in 15 cases (20.0%). However, the evaluation of lymph node density was hampered by the lack of use of contrast medium in the examinations. Right paratracheal region (2R) and those in the prevascular region (4L) were the most affected lymph nodes groups. Pleural and mediastinal changes found in CT2 included loculated pleural effusion in 1 case, increased pericardial thickness in 1 case, pleural thickness in 9, and calcified lymph nodes in 35 cases.

## 4. Discussion

The main limitations of the present study were small sample size, mediastinal evaluation (lymph nodes) without the use of contrast medium, and change in therapeutic regimen from three drugs to four drugs (ethambutol was included in 2014).

In Brazil, TB predominantly affects adults. The distribution of new TB cases by age and gender indicates a concentration of cases in the age group of 25–34 years with predominance of men [2, 9, 10]. In the group under study, no significant differences were observed in relation to gender, and their age varied between 18 and 77 years, with a concentration of cases between the 3rd and 4th decades.

Previous studies have reported that the percentage of patients with active TB and negative AARB testing varies between 21% and 47% of cases, and some studies have emphasized the loss of assay sensitivity as a possible cause [1–3]. From a clinical perspective, it is very difficult to establish a diagnosis based on the symptoms of TB. Clinical outcome is an important step in the formulation of a hypothesis. Although TB can be asymptomatic and only discoverable by using imaging tests, in most cases, this disease progresses with the development of symptoms including fever, weight loss, night sweats, and coughing. Alcantara et al. [11] highlight that anorexia, weight loss, and fever are significant outcomes in patients with pulmonary TB who seek reference healthcare services for TB, and they concluded that weight loss can be regarded as a TB predictor in patients with cough for more than two weeks and living in areas with high prevalence of TB. In our study, the main symptoms were consistent with those described in the literature, including cough in 78% of cases, asthenia—reported by patients with weakness—in 74% of cases, and weight loss in 68% of cases.

Early diagnosis is critical for TB control. Late diagnosis results in the ongoing transmission of TB in the community and in the development of severe and progressive forms of TB. Delay in the diagnosis of TB can cause more sequelae in the long term and has a greater impact on transmission and mortality [1, 12, 13]. Delay in identification of pulmonary TB cases has several reasons, including delay in the evaluation of patients with respiratory symptoms, lack of suspicion of TB, and loss of sensitivity of the most commonly used assay in the diagnosis—AARB in sputum. Several studies have indicated a long period of time between the onset of symptoms and diagnosis [1, 2, 12–15].

In our study, we found that duration of symptoms, defined as the interval between the onset of symptoms and the first consultation at the clinic, was too long, that is, up to 730 days. Several patients were referred after successive medical appointments; negative AARB test results in sputum as well as an incorrect diagnosis and treatment for pneumonia, which leads us to hypothesize that the main reasons are the lack of TB suspicion by physicians and a loss of sensitivity of sputum smear microscopy, which agrees with the results of previous studies [1, 2, 15]. We also analyzed the period between CT1 and the initiation of treatment. This period was also considered extensive (average of 51 days). On the basis of CT data, a decision on the initiation of TB treatment could have been taken during the period of diagnostic investigation. No previous studies have evaluated the period between the performance of CT and TB diagnosis.

Based on imaging tests, TB can manifest as multiple isolated or associated alterations that compromise the parenchyma, airways, pleura, and mediastinum, among other tissues [16].

In most cases, more than one lung segment is affected, and bilateral involvement is observed in approximately 88% of cases [3, 16, 17]. Most affected segments are the apical and posterior segments of the upper lobes and the apical segments of the lower lobes [2, 4, 17]. With regard to the length of the disease, we observed that multilobar and bilateral involvement is much more frequent than unilateral involvement, which may be due to the delay in the diagnosis. The most affected segments were the apical and posterior segments of the right upper lobe and the apical and posterior segments of the left upper lobe, findings in agreement with the results of previous studies [1, 2, 15].

The most common manifestation of post-primary TB results from the filling of the alveoli with dense material that replaces the alveolar air, and, radiographically, this manifestation is characterized by grouped nodular opacities and consolidations [5, 17, 18] with air bronchograms as frequent findings.

Architectural distortions were observed in 71% of cases, and these results corroborate those of previous studies [5–7, 16, 17].

Signs of bronchogenic dissemination of TB give the disease a peculiar characteristic. This aspect has been widely described in the literature as nodules in the airway space, centrilobular nodules, and tree-in-bud patterns when branched. In fact, they indicate granulomatous inflammatory changes at the level of the distal airways. Although not pathognomonic, these changes are very suggestive of TB, especially when analyzed within a favorable clinical epidemiological context [3, 5, 16, 19, 20]. This outcome was verified in our study in 93% of cases.

Airway involvement is frequently observed in active TB, is often associated with bronchogenic dissemination, and is characterized by increased thickness and dilation of the bronchial walls [5, 7, 21]. The higher frequency of these changes found in the present study is certainly associated with the extended period of disease activity.

Cavitary lesions are a sign of disease progression and a hallmark of reinfection of TB. Previous studies have found a clear association between cavitary lesions with increased wall thickness (mm to cm) and positive results for sputum smear microscopy [1, 16, 19, 20, 22]. In the present study, cavitary lesions were observed in 62% of cases—all with negative results for sputum smear microscopy—between 1.2 cm and 8.0 cm in diameter and wall thickness greater than 3 mm.

Lymph node involvement on CT has been reported in post-primary TB with a frequency between 15% and 43% in immunocompetent patients [5, 7, 22, 23]. In the present study, we found lymph nodes with a diameter greater than 1 cm in 20% of cases.

Pleural involvement in TB usually manifests 3 to 7 months after exposure to KB and is regarded as a late reaction to primary infection. Its frequency varies between 6% and 38% of cases. In most cases, pleural involvement is unilateral with small to intermediate volume and is easily visible on chest radiography. It can be the only radiographic manifestation of the disease in one-third of the cases, and CT can reveal the associated parenchymal changes in most cases [5, 7, 22]. In our study, pleural abnormalities were observed in 54% of cases and consisted of small-volume unilateral pleural effusion in 45% of cases and increased pleural thickness in 55%. That is, in patients with negative AARB testing, as in our study, the presence of pleural changes is more frequent than in patients with positive AARB testing.

In CT2, we identified a high frequency of changes considered sequelae and diagnosed them as architectural distortion in 92% of cases and cylindrical bronchiectasis in 86% of cases. Thin-walled cavitary lesions and reduced injury volume have been reported in several studies as radiographic and tomographic criteria for successful treatment of TB [16–18]. We observed that cavitary lesions with wall thickness of <3 mm did not disappear. Of note, we found that 48% of cases had persistent nodules between 1 and 3 cm in diameter. These surprising but rarely reported data deserve to be recognized by doctors and underscore the need for interpreting image data within a known clinical context, avoiding diagnostic and therapeutic procedures sometimes unnecessary in patients already treated. Certainly, the large number of sequelae found, notably those associated with bronchiectasis and residual nodules known as tuberculomas, may be associated with the long duration of the disease and the late diagnoses.

The main limitations of the present study were the small sample size and the mediastinal evaluation (lymph nodes) without the use of contrast medium. From an epidemiological point of view, the prevalence of TB is still high in Brazil, which is not the case with nontuberculous mycobacteria (NTM). However, it would have been interesting to jointly study another patient group with NTM for comparison of the image changes between them. CT certainly adds information, but its applicability in primary and secondary healthcare services can be considered another limitation.

## 5. Conclusion

It was possible to recognize a tomographic diagnostic pattern for active TB characterized by airway changes, which included increased wall thickness, dilation, and coalescence of bronchi associated with parenchymal changes identified as airway nodules configuring the tree-in-bud pattern, nodules between 1 and 3 cm in diameter, consolidation, architectural distortion, and cavitary lesions. There was agreement among the three radiologists in 85% of the cases. The high incidence of residual changes after treatment is most likely associated with late diagnosis.

## Figures and Tables

**Table 1:** Involvement of lobes and segments in 74 cases of active TB.

Right lung		Left lung	
Most affected segment	Number and percentage of cases	Most affected segment	Number and percentage of cases
Apical segment of the right upper lobe (1)	47 (63.5)	Apical-posterior segment of the left upper lobe (1/2)	55 (74.3)
Posterior segment of the right upper lobe (2)	49 (66.2)	Anterior segment of the left upper lobe (3)	32 (43.2)
Anterior segment of the right upper lobe (3)	34 (45.9)	Upper segment of the lingular lobe (4)	26 (35.1)
Lateral segment of the middle lobe (4)	21 (28.3)	Lower segment of the lingular lobe (5)	25 (33.7)
Medial segment of the middle lobe (5)	21 (28.3)	Apical segment of the left lower lobe (6)	39 (52.4)
Apical segment of the right lower lobe (6)	31 (41.8)	Anterior-medial segment of the left lower lobe (7/8)	21 (28.3)
Medial segment of the right lower lobe (7)	13 (17.5)	Lateral segment of the left lower lobe (9)	23 (31)
Anterior segment of the right lower lobe (8)	15 (20.2)	Posterior segment of the left lower lobe (10)	19 (25.6)
Lateral segment of the right lower lobe (9)	16 (21.6)		
Posterior segment of the right lower lobe (10)	15 (20.2)		

L, lobe; U, upper; R, right; Le, left; Lo, lower.

**Table 2:** Frequency of changes observed before treatment (CT1) and after treatment (CT2).

Changes	CT1 (number and percentage)	CT2 (number and percentage)
*Bronchial changes*		
Increased wall thickness	71 (95.9)	0
Dilation	69 (93.2)	64 (86.4)
*Parenchymal changes*		
Tree-in-bud pattern	69 (93.2)	04 (5.4)
Major nodules 1 to 3 cm in diameter^*∗*^	64 (86.4)	36 (48.6)
Consolidations	57 (77.0)	19 (25.6)
Air bronchograms	50 (67.5)	1 (1.3)
Architectural distortions	53 (71.6)	68 (91.8)
Cavitary lesions^*∗∗*^	46 (62.0)	12 (16.2)
Parenchymal calcifications	32 (43.2)	35 (47.2)
Ground-glass opacities^*∗∗∗*^	28 (37.8)	3 (4.0)
Air trapping	8 (10.8)	7 (9.4)

^*∗*^Of the 64 patients with nodules, one patient showed a halo sign and another showed an inverted halo sign.

^*∗∗*^The cavity wall was measured in the region with the largest thickness. The average thickness was 4.7 mm, with a median thickness of 4.45 mm and a standard deviation of 1.907.

^*∗∗∗*^Of the 28 patients with ground-glass opacities, 6 presented lung bleeding, which was characterized by hemoptoic expectoration in 5 cases and hemoptysis in 1 case.

## Additional Points

(i) Results of sputum smear microscopy were negative in approximately one-third of TB cases. (ii) Chest CT is helpful in the diagnosis of TB when sputum smear microscopy results are negative. (iii) In most of cases, the tree-in-bud pattern disappears after drug therapy. (iv) CT findings persist after TB treatment.
